# Therapeutic Effect and Mechanisms of Essential Oils in Mood Disorders: Interaction between the Nervous and Respiratory Systems

**DOI:** 10.3390/ijms22094844

**Published:** 2021-05-03

**Authors:** Timothy K. H. Fung, Benson W. M. Lau, Shirley P. C. Ngai, Hector W. H. Tsang

**Affiliations:** Department of Rehabilitation Sciences, The Hong Kong Polytechnic University, Kowloon, Hong Kong 999077, China; timothy.kh.fung@connect.polyu.hk (T.K.H.F.); benson.lau@polyu.edu.hk (B.W.M.L.); shirley.ngai@polyu.edu.hk (S.P.C.N.)

**Keywords:** inhalation therapy, essential oil, anti-depression, anxiolytic

## Abstract

Essential oils (EOs) are extracted from plants and contain active components with therapeutic effects. Evidence shows that various types of EOs have a wide range of health benefits. In our previous studies, the potential of lavender EO for prevention and even treatment of depression and anxiety symptoms was demonstrated. The favourable outcomes may be due to multiple mechanisms, including the regulation of monoamine level, the induction of neurotrophic factor expression, the regulation of the endocrine system and the promotion of neurogenesis. The molecules of EOs may reach the brain and exert an effect through two distinctive pathways, namely, the olfactory system and the respiratory system. After inhalation, the molecules of the EOs would either act directly on the olfactory mucosa or pass into the respiratory tract. These two delivery pathways suggest different underlying mechanisms of action. Different sets of responses would be triggered, such as increased neurogenesis, regulation of hormonal levels, activation of different brain regions, and alteration in blood biochemistry, which would ultimately affect both mood and emotion. In this review, we will discuss the clinical effects of EOs on mood regulation and emotional disturbances as well as the cellular and molecular mechanisms of action. Emphasis will be put on the interaction between the respiratory and central nervous system and the involved potential mechanisms. Further evidence is needed to support the use of EOs in the clinical treatment of mood disturbances. Exploration of the underlying mechanisms may provide insight into the future therapeutic use of EO components treatment of psychiatric and physical symptoms.

## 1. Introduction

Essential oil (EO), as the main component used in inhalation therapy, has been widely investigated for its therapeutic effects. Evidence indicates that EOs can successfully reduce anxiety and relieve pain when combined with conventional treatment [1,2]. EOs can be administered through oral consumption, direct skin contact, or inhalation [3]. Among all different administration routes, inhalation is the most commonly adopted method. In fact, EOs from different plant extracts have been studied to demonstrate different effects, with lavender and bergamot EOs being the most widely used ones for relaxation in either single use or mixed use with reported pharmacodynamic interactions [4]. It has been shown that the different positive effects were linked to specific constituents in EOs [5,6]. Therefore, the combined use of EOs consisting of different molecular compounds, is able to maximise the therapeutic effect [7,8].

While the clinical effectiveness of EOs on depression and mood disturbances has already been proven, the underlying mechanisms of the therapeutic effects still remain elusive. In this review, we will discuss the biological routes of the volatile EO molecules upon entering the body and the possible cellular and molecular mechanisms underlying their therapeutic effects.

## 2. Therapeutic Effects of EOs

Our previous studies have shown EOs to be effective in alleviating symptoms of depression, anxiety, and stress in adults both below and over 60 years of age, respectively [9,10]. Table 1 summarises the effect of EOs in human studies. For instance, the inhalation of lavender and chamomile EOs was found to decrease levels of depression, anxiety, and stress in older adults. It was suggested that the anxiolytic and antidepressant effects could be associated with the suppression of the activity of the sympathetic nervous system [11]. In addition, beneficial effects on physiological health, such as reduction of blood pressure levels and respiratory rate during panic attacks after the inhalation of lavender EO have been reported [12].

Moreover, EOs have the potential to relieve depression and secondary depressive symptoms arising from different types of chronic conditions, like anxiety disorders and dementia [13,14,15,16]. Fayazi and co-workers have shown that patients inhaling lavender EO before undergoing heart and abdominal surgeries reported less anxiety, suggesting the potential anxiolytic effect of the EO [17].

The therapeutic effects of EOs have also been proven in animal studies (Table 2). Behaviour tests, including open field test (OFT), elevated plus maze test (EPM), and forced swimming test (FST) are commonly used to examine the anxiety and depression levels of animals [28,29]. After inhalation of lavender EO, both locomotor activity in OFT and open arm timing in EPM were shown to increase, implicating the anxiolytic effect of lavender EO [30,31], whereas reduction of immobility in FST implicated an antidepressant effect [22]. In addition, lemon and ylang-ylang EOs were also found to be effective in reducing anxiety symptoms [32,33].

Animal studies have helped in the investigation of the underlying mechanisms of EOs. The anxiolytic effects of EOs were found to be associated with increasing serotonin (5-HT) level or dopamine (DA) level [31,32,34], while the antidepressant effect was correlated with an increase of brain-derived neurotrophic factor (BDNF) expression level [35]. Apart from lavender, lemon, perilla, and asarum EOs have also shown similar results, including reduced immobility time in FST, and increased 5-HT and DA levels [35,36,37]. Moreover, Zhang and colleagues reported that ylang-ylang EO activated the mitogen-activated protein kinase (MAPK) signalling pathway, associated with 5-HT expression and cellular growth [33].

Although most of the EOs can interact with a range of neurotransmitter pathways, such as noradrenergic, 5-HTergic, γ-aminobutyric acid (GABA)-ergic and DAergic systems [38], the effect of the EOs mainly depends on the effectiveness of their active compounds. It has been reported that some of the major compounds of EOs, such as linalool, limonene, benzyl benzoate, and benzyl alcohol have exerted anxiolytic and antidepressant effects [39]. The underlying mechanism of action of specific compounds has been investigated in pharmacology studies. Benzyl benzoate in ylang-ylang activated the 5-HTergic and DAergic pathways, which had an effect on anxiety [39]. Other study results indicated that linalool and β-pinene produced an effect through interaction with the GABAergic pathway. As lavender and bergamot EOs both contain linalool, they can act on GABA receptors to claim an anxiolytic and antidepressant effect [40]. Furthermore, cinnamon EO also has an anxiolytic effect by inhibiting the release of pro-inflammatory cytokines [38]. Sweet orange, rose and lavender EOs produce a sedative effect through interacting with the hypothalamic–pituitary–adrenal (HPA) axis to reduce cortisol concentration in serum [38]. The effectiveness of EOs may vary depending on the differences in concentration. The combination of different chemical compounds in EOs may interact with different neurotransmitter pathways resulting in a wide range of therapeutic effects. In addition, odorant molecules can also interact with olfactory receptors to trigger smell signal transmission and, thus, enhance the positive effect on mood. Different odorant molecules may induce different unique electrical signalling patterns in the olfactory system, thereby inducing different modulatory effects on mood. For further elaboration, see sections below.

## 3. Absorption of EO Molecules through Inhalation (Mechanisms behind the Effect of EOs on Brain)

**Figure 1:**
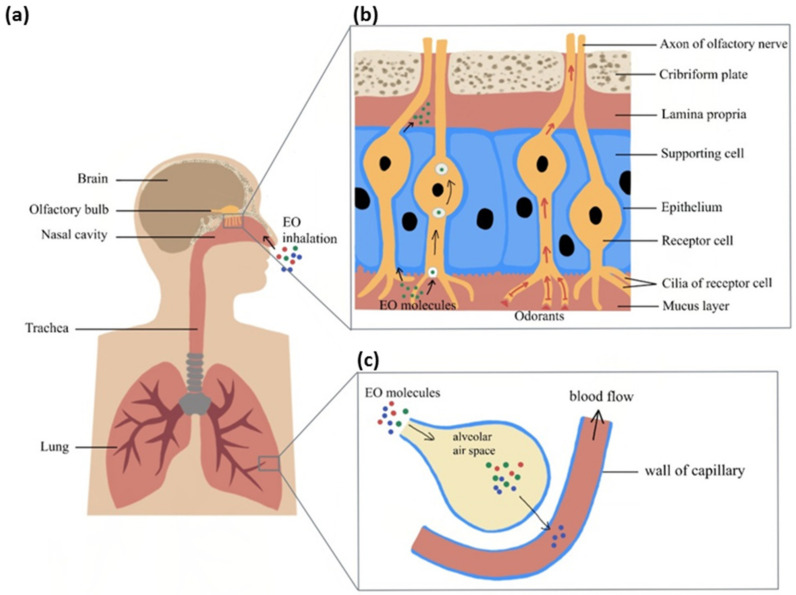
Inhaled EO response delivery to brain through the respiratory and olfactory systems: (**a**) Inhaled EO passes through the nasal cavity and reaches the olfactory system or respiratory system (**b**) Overview of EO molecules’ delivery pathway in the olfactory system (**c**) Overview of EO molecules crossing the air–blood barrier to reach the circulatory system.

EOs are composed of a variety of volatile chemical constituents. To facilitate the absorption of EOs, a novel administrative method utilising nanotechnology has been developed. By encapsulating EOs into nanoparticles, the uptake and effect of them can be enhanced. The inhaled EO molecules will be delivered to the brain region via different pathways according to their molecular sizes. The possible inhalation delivery pathway involves both the olfactory and respiratory systems (Figure 1). The olfactory system begins with the nasal cavity connected to the olfactory bulb and plays an important role in odorant signal transmission. The olfactory system is located close to the brain and connects to several brain areas, including the hypothalamus and the hippocampus. Some fine chemical molecules can pass through the axon of the sensory neuron cells or olfactory mucosa and be directly delivered to the central nervous system (CNS) to change the emotional response. On the other hand, the respiratory system is mainly responsible for gaseous exchange. Vapour molecules can reach different regions of the respiratory system by diffusion. For example, molecules dissolve into the respiratory epithelium and diffuse to the alveoli and blood to then be transported in the bloodstream to the brain.

There are three potential basic mechanisms enabling EOs to influence brain functioning. The first mechanism involves the activation of nasal olfactory chemoreceptors and the subsequent effect of olfactory signals on the brain. The olfactory system is unique among the sensory systems for having direct anatomical and functional links with the limbic system. Thus, olfactory stimuli can have a strong effect on mood. The second putative mechanism of action is direct penetration of EO molecules via the olfactory nerve into connected brain areas and the induction of cellular and molecular events. The third potential pathway is the alveolar absorption of EO molecules into the blood circulation, crossing the blood–brain barrier (BBB) to interact with specific brain regions.

### 3.1. Transmission of Olfactory Odorant Signal (Through Activation of Nasal Olfactory Chemoreceptors)

The key in identifying odour and triggering the corresponding effect lies in the olfactory sensory neurons (OSN). Humans have around 400 functional genes coding for OSN receptors, with each OSN expressing a specific type of odorant receptor [48,49]. Electrophysiological research in animal studies has shown that a particular odorant could activate a single or a unique combination of OSN receptors with different affinities to generate a specific signal. Hence, odours can be identified and discriminated. Different types of OSNs are distributed to the olfactory epithelial layer. When EO is inhaled, the sense of smell is perceived following the activation of nasal olfactory chemoreceptors by the volatile odorant molecules. The odorant molecules pass through the olfactory epithelium and bind to the dendrite receptors on the OSNs to generate signals by inducing an action potential. The axons from the same type of OSNs project and converge to its corresponding glomerulus cell in the olfactory bulb [50]. Each glomerulus input is associated with specific types of mitral and tufted cells. The dendrites of the glomerulus then transmit the excitatory input to the connected mitral and tufted cells. Finally, the specific transmitted signals are delivered to the pyramidal neurons in the olfactory cortex where the signal can further stimulate the corresponding regions of the brain [51].

**Figure 2:**
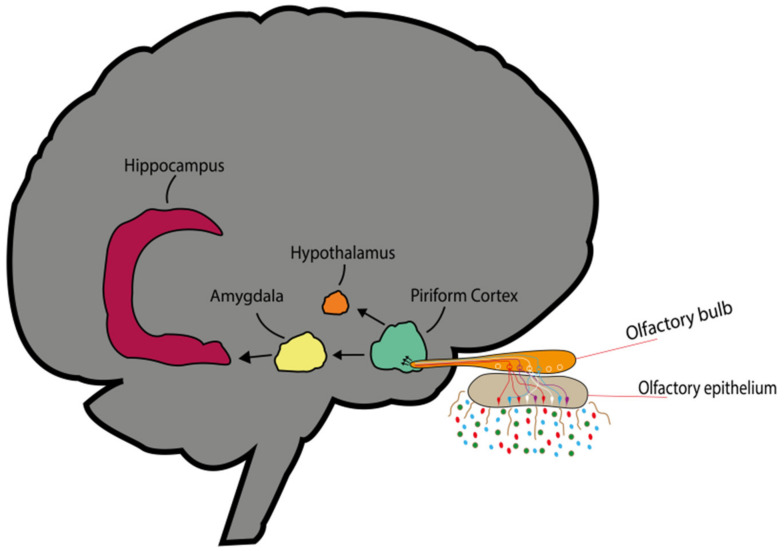
Inhaled odour molecule activating unique OSN receptors. The axon of the OSN converges to its corresponding glomerulus and each glomerulus input to its associated mitral and tufted cells. The odorant signal is further transmitted into the olfactory cortex where it stimulates the corresponding regions of the limbic system.

Some neurophysiological studies have examined the olfactory cortex in relation to brain mapping to identify the specific function of the activated brain region [52,53]. The olfactory cortex includes several regions, such as the piriform cortex, the olfactory tubercle, and the entorhinal cortex. Each region can further project information to the limbic system, i.e., the amygdala, the hippocampus, and the hypothalamus (Figure 2). The olfactory signal, unlike other sensory systems, can project the signal through the ipsilateral axon. The signal can then be directly transmitted to the cortex without including the thalamus. It realises a highly specific, and direct connection between odour, memory, emotion, and endocrine function. Some studies have been investigating odorant-evoked signals in the olfactory cortex [54,55]. The piriform cortex and orbitofrontal cortex have the function of odour identification and integration of olfactory information. Furthermore, the piriform cortex and entorhinal cortex transmit signal information to the amygdala and the hippocampus. These neuronal connections provide the basis for the interaction of memory and mood. The hippocampus plays a role in odour memory formation [56], while the amygdala is responsible for the processing of the emotional response and the controlling of scent intensity. The piriform cortex can also project to the hypothalamus, which is essential for hormone secretion. In fact, the HPA axis, also known as the stress response system [57], is responsible for triggering responses of the autonomic nervous system, including heart rate, blood pressure, and breathing rate [58]. Thus, the scent molecules of the EOs, through activation of smell signals in the brain, can have a therapeutic effect on mood.

On the other hand, several odorants evoke electrical signal to modulate the specific physiological effects relating to mood and behavioural responses [59,60]. Olfactory bulb mapping has been investigated previously to reveal the relationship between olfactory signal transmission and behavioural responses in the olfactory cortex. Different types of OSNs converge to their corresponding glomeruli in the olfactory bulb, which is divided into dorsal and ventral sides. One study reported that the dorsal region of the olfactory bulb triggered initial fear response, while the ventral region triggered fear memory [61] through stimulating the hippocampus. These studies provided evidence for the relationship among odorants, behavioural response, and brain activation related to emotion and cognition. However, the mapping remains elusive due to the complexity of the network. To further confirm if exposure to EOs would indeed activate these regions, activation studies on c-Fos expression in animals and fMRI studies in humans are needed.

### 3.2. Chemical Transport of Molecules (Direct Penetration of EO Molecules via Neuronal Pathway)

Apart from olfactory signal transmission, EOs can also exert an effect on mood through intra and extracellular transport. After being inhaled into the nasal cavity, the small volatile compounds may pass through the neuronal network of the olfactory system and directly arrive at the brain [62,63]. Intracellular transport is initiated when the inhaled molecular compounds bind to the surface receptors of the target neurons. The molecular compounds can prompt the internalisation of the receptors and, consequently, initiate receptor-mediated endocytosis. Subsequently, the molecules end up in the olfactory cortex via anterograde transport and further interact with the hippocampus and the amygdala. The two cranial nerves, i.e., the olfactory nerve and the trigeminal nerve, are the nerves involved in the anterograde transport. The molecules entering the OSN are transported by endosomes to the olfactory bulb along the axons. On the other hand, the axon of trigeminal sensory neurons is connected to the pons. The pons is part of the hindbrain and is essential in relaying information to the cerebellum. It may also project to the medulla to regulate and control breathing rate.

Another form of transport is extracellular transport in which the molecular compound passes through the paracellular cleft between the supporting cell and OSN to get into the lamina propria (connective tissues) via fluid movement [64,65]. After reaching the lamina propria, the molecules will be further transported externally along the axons in the perineural space and ultimately reach the brain parenchyma. Subsequently, the molecules get across the BBB and blood–cerebrospinal fluid barrier to reach different brain regions [66].

Furthermore, the EO molecule is able to interact with the neurotransmitter receptor in such a way that the transient receptor potential channels (TRP), GABA, 5-HT, and DA receptors produce an anxiolytic or antidepressant effect.

### 3.3. Respiratory System and Central Nervous System (Alveolar Absorption of EO Molecules into the Blood Circulation with Subsequent Effect on Brain)

Some studies have proposed that EOs could be absorbed through the respiratory system [30,67,68]. Together with room air, the inhaled EO molecules would be delivered through the respiratory tract and transported to the alveolar sacs [69,70], although only soluble molecules would be able to cross the air–blood barrier. EOs are mixtures of many molecular compounds, with some being polar and others nonpolar. The majority of EO components are of the lipid-soluble terpene family, i.e., lipophilic and hydrophobic in nature. With that said, some EO components have been reported as being soluble in water. Alveolar diffusion is the main pathway for molecular delivery to the systemic circulation. EO molecules that are lipophilic in nature can be transported across the BBB to the CNS [63]. Previous studies have shown that the systemic transport of the volatile EO molecules can activate the CNS [71], though the activation may be due to crossing the BBB directly or certain secondary effects. The activation of the CNS induces positive psychological and physiological effects.

Breathing is an automatic response controlled by the vegetative nervous system of the brain. Meanwhile, respiration can be altered by exposure to stress, which would activate the limbic system and trigger physiological responses. Physiological responses can represent emotional responses [72]. Thus, respiration has a unique correlation with emotion [73]. On the other hand, respiratory health is also linked to brain function. Evidence for this is that pulmonary diseases have proven to affect brain cell growth via brain–lung crosstalk [74]. Systemic circulation includes the transport of lung-induced inflammation mediators, which trigger adaptive responses in the brain [75]. Moreover, lung function declination also reduces oxygen supply in the body, especially in the brain, causing brain injury, and neuropsychological and neurobehavioural disorders, such as depression and anxiety [76]. Conversely, the relationship between respiratory health and mental health is a closely knit one.

## 4. Effect of EO on Cellular/Molecular Events

In this section, the potential molecular and cellular mechanisms underlying the therapeutic effect of different EOs in both human and animal studies are discussed. Unlike humans, animals cannot express subjective emotional states. Whereas in human studies the severity of depression and anxiety can be assessed via questionnaires or interviews [77], the examination of depression- and anxiety-like state in animals requires standardised tests, such as FST and OFT [78,79]. The underlying mechanisms of EOs driving depression and anxiety-like behaviour will be elaborated on below.

### 4.1. Regulation of Monoamines

According to the 5-HT hypothesis of depression [80], the signs and symptoms of depression are the result of 5-HT deprivation. The 5-HT hypothesis is currently the accepted hypothesis with regard to the pathophysiology of depression.

The effect of EOs on the 5-HTergic system has been demonstrated in various studies. In one clinical study, community-dwelling older adults were involved in therapeutic massage/inhalation treatment combining lavender, sweet orange, and bergamot EOs for 8 weeks. After the treatment, depressive symptoms decreased compared to the baseline assessment, as measured by the geriatric depression scale and the patient health questionnaire-9. The decrease of symptoms was associated with an increase of plasma 5-HT in the EO-treated group [81]. In another study, which used compound anshen EO, the inhalation of the EO improved sleep quality and promoted 5-HT increase in experimental mice [82]. An increase of 5-HT-expressing neurons in the dorsal raphe nucleus was found in animals that inhaled EO from asarum heterotropoides for 3 h [45], suggesting that the positive effect of EO inhalation may occur in both acute and chronic conditions.

Bergamot EO (BEO), which has shown to have both anxiolytic and antidepressant effects, is a typical choice adopted in the management of depression [83]. In an acute animal experiment [83], when BEO was used in combination with WAY-100635, an antagonist of 5-HT1A, a synergistic effect was observed in terms of anxiety-like behaviour. In this particular experiment, it is possible that WAY-100635 augmented the anxiolytic effect of BEO by antagonising the presynaptic 5-HT1A autoreceptors, which increased the release of 5-HT in the synapse. While only the acute effect of BEO was tested in the experiment, to understand its full potential with regard to 5-HT receptors, the chronic effect of BEO on the expression and function of presynaptic 5-HT1A receptors would need to be examined.

Ylang-ylang EO (YYO) is another commonly used EO for relaxation, depression, and anxiety disorders [39]. Exposure to YYO has been shown to increase 5-HT concentration in the hippocampus, which is associated with the suppression of the ERK1/2/CREB pathway and ERK/CREB phosphorylation [39], usually activated following exposure to stress. While, in general, EOs have been associated with an increased release of 5-HT, the detailed mechanisms, including their influence on 5-HT receptors and signalling pathways, still remain elusive.

Although the 5-HT hypothesis is the dominant hypothesis in terms of the pathology of depression, the roles of other monoamines, in particular DA, have also been proven. Anhedonia, a core symptom of mood disorder characterised by lack of interest, is closely linked to the mesolimbic DAergic system [84,85]. This circuit consists of the ventral tegmental area (VTA) and the nucleus accumbens (NAc), with the former region innervating the latter with DAergic input. The circuit is associated with the rewarding effects of food, sex, and drugs of abuse. If key proteins, including BDNF and cAMP-response element binding (CREB) protein are manipulated, rodents will display behavioural phenotypes similar to anhedonia. Thus, circuitry may play an important role in the pathophysiology of depression. Both basic and clinical studies have shown that deficits in the DAergic system were evident in depression. For instance, a study on the antidepressant effect of vanillin showed increased levels of both 5-HT and dopamine in brain tissues [86], whereas results from another study showed that lemon EO, though having an antidepressant effect, accelerated the turnover of DA in the hippocampus. When apomorphine, a nonselective DA receptor agonist, was administered, the anxiolytic effect of lemon EO significantly reduced. The DAergic system is likely participating in the therapeutic effect of EOs; however, further confirmatory tests are needed to specify the mediatory role of DA.

### 4.2. Neurogenesis and Neurotrophic Factors

In the past two decades, the speculation that depression is caused by a deficiency in neurogenesis was widespread [87]. This speculation originated in the observation that chronic antidepressant treatment promoted neurogenesis in the hippocampus of experimental rodents [88], and stress, a known risk factor in developing psychiatric illnesses, was shown to reduce neurogenesis [89]. In animal models of depression, suppressed neurogenesis is usually found to be a common feature [90]. When neurogenesis is suppressed by x-irradiation, the therapeutic effect of antidepressants is abolished [91]. These observations collectively suggest that the production of new neurons is likely involved in the pathophysiology of depression and affects the efficacy of antidepressant treatments. Recently, some reports have shown that the therapeutic benefits of EOs may be due to a pro-neurogenic effect [35,92]. In an animal study that employed corticosterone to induce depression-like behaviour, chronic exposure to lavender EO prevented such negative effects of depression as suppressed neurogenesis, suppressed dendritic growth of immature neurons and decreased serum BDNF levels [35]. Another study, using a chronic unpredictable mild stress model to induce depression-like behaviour in animals showed that exposure to musk stimulated neurogenesis and reduced the neuronal apoptosis in the hippocampus, which is associated with the upregulation of hippocampal BDNF levels [92].

Neurotrophins are a class of growth factors that promote the development of neurons and enhance neuroplasticity [93]. Among the different neurotrophins, BDNF has received the most attention; hence, the neurotrophic hypothesis of depression. In this hypothesis, the etiology of depression is related to the deprivation of neurotrophic factors caused by exposure to stress and decrease in neural plasticity [94]. The involvement of BDNF in depression is based on its function as well as evidence from clinical studies. BDNF is the most abundant neurotrophic factor in the brain, promoting the growth of the nervous system and enhancing neural plasticity. In terms of depression, the serum level of BDNF was found to decrease in antidepressant-naive patients, whereas treatment with antidepressants was found to reverse this decrease [95]. In accordance with the above hypothesis, studies on EOs and neurotrophic factors have mainly focused on the expression levels of BDNF.

In a clinical study, women whose children were diagnosed with attention deficit and hyperactivity disorder were recruited, assuming that this particular population suffered from considerable stress affecting their mental health [96]. After a 4-week treatment program with EOs, the level of anxiety and depression of the subjects decreased, while the plasma BDNF level (reflecting the brain tissue level of BDNF) significantly increased. In other animal models of depression assessing the effect of EOs, similar findings have been observed as well. In an animal model of depression induced by chronic mild stress, treatment with musk relieved depression-like behaviour, which was associated with an increase in BDNF expression in the hippocampus [92]. Another study, which used a mixture of lemon and rosemary EOs for 2 months on mice, also showed a marginally higher level of BDNF along with the improvement of cognitive functions [97]. In terms of the signalling pathway, one study tested whether d-limonene, a major component of orange EOs shown to have an antidepressant effect, would promote neuronal development [98]. After treatment with d-limonene, PC12m3 cells showed enhanced neurite outgrowth along with the activation of the p38MAPK pathway. When treated with p38MAPK inhibitor, the effect of d-limonene was suppressed. Compared to the MAPK pathway, ERK and JNK were weakly activated by d-limonene. In general, there are both basic and clinical studies in support of the therapeutic effects of EOs with regard to the regulation of neurotrophic factors. On the other hand, active components in EOs may affect neuronal growth via the regulation of neurotrophic factors and the direct activation of trophic pathways. Further mechanistic studies on the signalling pathway and the regulation of neurotrophic factors are necessary to better understand the processes behind the beneficial effects of EOs.

### 4.3. Regulation of the Neuroendocrine System

It is believed that regulation of hormonal levels is a potential underlying mechanism explaining the effect of EOs, for instance, in the treatment of premenstrual syndrome [99] and menopausal disorder [100,101]. Since stress is a risk factor in depression and anxiety disorders, stress hormone cortisol has been the focus of studies examining the antidepressant and anxiolytic effects of EOs [102]. In human studies, acute exposure to lavender EO has been shown to decrease salivary and serum cortisol levels [103,104]. Simultaneously, findings indicated increased relaxation in subjects, including improved coronary flow velocity [104]. The cortisol-suppressing effect was reported in adults [105], pregnant women [106], mothers of children with developmental dysfunction [96], young children [107], as well as patients undergoing chemotherapy [108]. Apart from lavender, other EOs were also shown to affect cortisol levels, including bergamot [23,109] and grapefruit seed [110].

Animal studies provide cues on the alteration of the HPA axis resulting from exposure to EOs. Acute inhalation of bergamot EO attenuated the rise of corticosterone level response to stress induced by exposure to EPM test, which implies an effect of bergamot on the HPA axis [111]. Inhalation of an active component of nardostachys chinensis, an herbal tranquiliser, was also shown to reduce restraint-induced stress responses and attenuate the increase in corticosterone levels [112]. Similar findings were reported in a study that used musk for inhalation [96] and another one that used methyl jasmonate (MJ) [113]. However, another animal study brought out that the corticotrophin-releasing hormone (CRH), which regulates the release of the adrenocorticotropic hormone (ACTH) and cortisol in the HPA axis, could increase via the inhalation of EOs from asarum heterotropoides [45].

Interestingly, the effect of EOs on depression-like and anxiety-like behaviour has been linked to genetic predisposition of temperament in sheep [114]. Upon exposure to lavender EO, anxiety-like behaviour as well as the serum cortisol level of sheep with calm traits showed a decrease, whereas sheep with nervous traits showed an increase in both anxiety-like behaviour and serum cortisol level.

### 4.4. Other Possible Mechanisms: Oxidative Stress and Inflammation

The growing understanding of the pathophysiology of depression and anxiety disorders allows us to link certain effects of EOs to mood and emotion. Oxidative stress, which is brought about by reactive oxygen species (ROS), has an important role in neurodegeneration [115]. Due to the requirement of high oxygen level for proper functioning, the CNS is susceptible to ROS insults. Downstream signalling, including pro-inflammatory signalling and cellular apoptosis is triggered by ROS, and the undesirable consequences are believed to be potential causes of depression [116]. Based on the assumption that ROS and inflammatory signalling have a role to play in depression, EOs may be beneficial in depression due to their antioxidant and anti-inflammatory properties. For instance, exposure to lavender and rosemary EOs was found to reduce free radical scavenging activity (FRSA), which further prevents the detrimental effects of oxidative stress [103]. In addition, cinnamomum cassia presl (CC-EO), the active component of cinamaldehyde EO also possesses antioxidant properties [117]. Exposure to MJ was effective in preventing depression-like behaviour induced by lipopolysaccharide (LPS) and attenuated the increase of malondialdehyde (MDA), glutathione (GSH), and tumour necrosis factor-alpha (TNF-α) in mice [113]. Though the antioxidant and anti-inflammatory properties of EOs are well-recognised, most of the relevant studies have focused on disorders related to ROS (e.g., skin disorders and wound healing) as well as immune disorders. Further studies on the role of ROS and inflammation in psychiatric disorders are needed to correlate oxidative stress and inflammation with the therapeutic mechanisms of EOs in terms of mood disturbances.

## 5. Future Studies

To facilitate the use of EOs in clinical practice when treating depression and anxiety disorders, further large-scale clinical trials are required to confirm the effectiveness and efficacy of them. Although various studies have illustrated that certain EOs possess antidepressant and anxiolytic effects [15,118], the choice of EOs is not standardised. As the active ingredients may vary among EOs due to non-standardised manufacturing processes, clinical trials with large sample size and standardised EOs as treatment agents are required.

Up till now, mechanistic studies exploring the molecular signalling pathways of EOs remain scarce. From a pharmacological perspective, the active components of EOs should be identified for elucidation of the background mechanisms. For instance, linalool has been identified as the major component in lavender EO [119]. The identification of potential active compounds would facilitate conducting biological assays. Identifying the active compound with therapeutic effect on behaviour would also allow the studying of the signalling mechanisms. Both animal and cellular studies would be valuable for further exploration, provided that standardisation of a particular type of EO is available.

## 6. Conclusions

**Figure 3:**
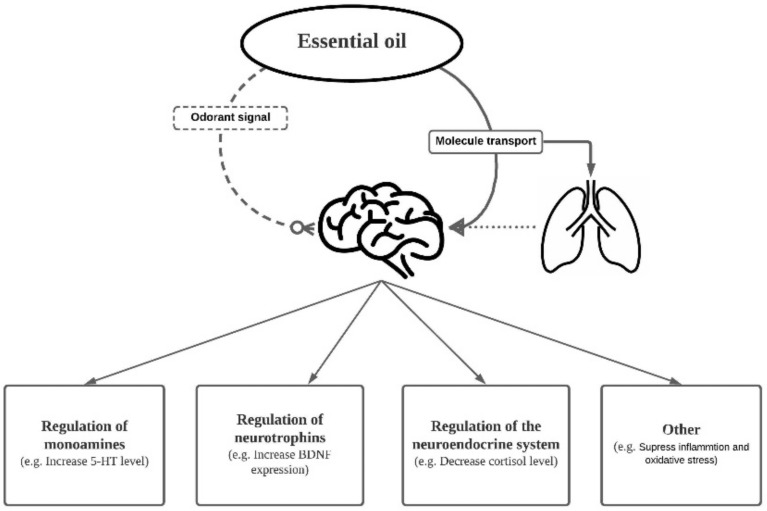
Summary of the EO delivery pathway in connection to the respiratory system and central nervous system. EO molecules exert an effect on mood disturbances via different possible routes, including regulation of monoamines, neurotrophic factors, and the neuroendocrine system, which are all potential mechanisms underlying the therapeutic effects.

In this review, we have discussed the antidepressant and anxiolytic effects of EOs in both human clinical and animal studies. The interplay between the respiratory system and CNS was also touched upon. Through the interconnection of the two systems, EO molecules exert an effect on mood disturbances via different possible routes. Cellular and molecular events, including alteration of monoamines, neurotrophic factors and neurogenesis are potential mechanisms underlying the therapeutic effects of EOs (Figure 3). In future studies, isolation of effective components of EOs would be essential to dissect the precise underlying action. This would not only reveal the background mechanisms but also suggest new targets for treatment of major depression and anxiety disorders.

## Figures and Tables

**Table 1:** Summary of clinical effects of EO inhalation on depression/anxiety disorders in human studies.

EO(s) Scientific Name (Common Name)	Author (Year)	Results	
*Lavandula*			
*Lavandula angustifolia* (Lavender)	Burnett et al. (2004)	Anxiolytic effect	[8]
	Lehrner et al. (2005)	Reduced anxiety; positive effect on mood; higher level of calmness	[18]
	Fayazi et al. (2011)	Anxiolytic effect	[17]
	Senturk et al. (2018)	Anxiolytic effect	[19]
	Karan et al. (2019)	Blood pressure control; anxiolytic effect; respiratory relaxation	[12]
	Ebrahimi et al. (2021)	Antidepressant effect; anxiolytic effect; reduced stress	[20]
*Citrus*			
*Citrus sinensis* (Sweet orange)	Lehrner et al. (2005)	Anxiolytic effect; positive effect on mood	[18]
	Goes et al. (2012)	Anxiolytic effect	[21]
*Citrus junos* (Yuzu)	Matsumoto et al. (2014)	Anxiolytic effect; antidepressant effect	[22]
*Citrus bergamia* (Bergamot)	Watanabe et al. (2015)	Reduced salivary cortisol level	[23]
*Matricaria*			
*Matricaria chamomilla* (Chamomile)	McKay et al. (2006)	Antidepressant effect; anxiolytic effect	[24]
	Ebrahimi et al. (2021)	Antidepressant effect; anxiolytic effect; reduced stress	[20]
*Salvia*			
*Salvia rosmarinus* (Rosemary)	Burnett et al. (2004)	Anxiolytic effect	[8]
*Salvia officinalis* (Sage)	Muss et al. (2010	Positive effect on mood	[25]
*Salvia lavandulaefolia* (Spanish sage)	Muss et al. (2010)	Positive effect on mood	[25]
Mixture			
*Lavandula angustifolia* (Lavender) + *Rosa damascena* (Damascus Rose)	Conrad et al. (2012)	Anxiolytic effect; antidepressant effect	[26]
*Lavandula angustifolia* (Lavender) *Cananga odorata* (Ylang-ylang) + *Citrus aurantium* (Neroli)	Song et al. (2017)	Anxiolytic effect	[27]

**Table 2:** Summary of pre-clinical examinations of EO inhalation on depression and anxiety-like symptoms in animal models.

EO(s) Scientific Name (Common Name)	Author (Year)	Animal	Behaviour Outcome (a)	Secondary Outcome (b)	Results	
*Lavandula*						
*Lavandula angustifolia* (Lavender)	Chioca et al. (2013)	Mice	MBT, OFT		(a) Increase in locomotor activity (b) -	[30]
	Chioca et al. (2013)	Mice	EPM	5-HT	(a) Increase in open arm timing (b) Increase of 5-HT level	[31]
	Coelho et al. (2014)	Rats	CFT	c-Fos	(a) Decrease in freezing response (b) Increase in c-Fos expression	[41]
	Sanchez-Vidana et al. (2019)	Rats	OFT, FST	DCX, BDNF	(a) Increase in locomotor activity; decrease in immobility timing (b) Increase in DCX expression and BDNF level	[35]
*Citrus*						
*Citrus limon* (Lemon)	Komiya et al. (2006)	Mice	EPM, OFT FST	DA, 5-HT	(a) Increase in open arm timing and locomotor activity; decrease in immobility timing (b) Increase of 5-HT and DA levels	[32]
*Citrus sinensis* (Sweet orange)	Hocayen et al. (2019)	Mice	MBT, OFT, Light/dark test	NADPH-d	(a) Increase in locomotor activity and spending time in bright area (b) Decrease of NADPH cells	[42]
Other						
*Acorus gramineus* (Japanese sweet flag)	Koo et al. (2003)	Mice		NADPH-d	(a) - (b) Decrease of NADPH cells	[43]
*Perilla frutescens* (Perilla)	Ji et al. (2014)	Mice	OFT, FST, TST	5-HT, 5-HIAA	(a) Decrease in immobility timing (b) Increase of 5-HT and 5-HIAA levels	[34]
*Coriandrum sativum* (Coriander)	Cioanca et al. (2014)	Rats	EPM, FST	GSH	(a) Increase in open arm timing; decrease in immobility timing (b) Increase of GSH	[44]
*Asarum caudatum* (Asarum)	Park et al. (2015)	Mice	FST, TST	CRF, 5-HT	(a) Decrease in immobility timing (b) Decrease of CRF; increase of 5-HT level	[45]
*Rosa disambiguation* (Rose)	Villareal et al. (2017)	Rats	EPM		(a) Increase in open arm timing (b) -	[46]
*Rosmarinus officinalis* (Rosemary)	Villareal et al. (2017)	Mice	TST	DA, Cort	(a) Decrease in immobility timing (b) Decrease of serum Cort level and increase of brain DA levels	[47]
*Cananga odorata* (Ylang ylang)	Zhang et al. (2018)	Mice	EPM	5-HT	(a) Increase in open arm timing and locomotor activity (b) Increase of 5-HT level	[33]

Abbrev. FST = Forced swimming test; OFT = Open field test; TST = Tail suspension test; EPM = Elevated plus maze test; MBT = Marble burying test; CFT = Contextual fear-conditioning test; GSH = Glutathione; NADPH-d = Nicotinamide adenine dinucleotide phosphate diaphorase; CRF = Corticotropin-releasing factor; 5–HT = Serotonin; 5-HIAA = 5-Hydroxyindoleacetic acid; DA = Dopamine; BDNF = Brain-derived neurotrophic factor; DCX = Doublecortin; c-Fos = Cellular oncogene fos; Cort = corticosterone.

## Data Availability

Not applicable.

## Abbreviations

5-HT	serotonin
ACTH	adrenocorticotropic hormone
BBB	blood–brain barrier
BDNF	brain-derived neurotrophic factor
BEO	bergamot EO
CC-EO	cinnamomum cassia presl
CNS	central nervous system
CREB	cAMP-response element binding
CRH	corticotrophin-releasing hormone
DA	dopamine
EO	essential oil
EPM	elevated plus maze test
FRSA	free radical scavenging activity
FST	forced swimming test
GABA	γ-aminobutyric acid
GSH	glutathione
HPA axis	hypothalamic–pituitary–adrenal axis
LPS	lipopolysaccharide
MAPK	mitogen-activated protein kinase
MDA	malondialdehyde
MJ	methyl jasmonate
NAc	nucleus accumbens
OFT	open field test
OSN	olfactory sensory neuron
ROS	reactive oxygen species
TNF-α	tumour necrosis factor-alpha
TRP	transient receptor potential channels
VTA	ventral tegmental area
YYO	ylang-ylang EO
